# Psychometric properties of the Vietnamese version of the 12-item HIV stigma scale (HSS-12) among people living with HIV in Vietnam

**DOI:** 10.1186/s40359-026-04833-7

**Published:** 2026-05-25

**Authors:** Cao Nguyen Hoai Thuong, Nguyen Vu Anh Thu, Bui Hong Cam, Le Minh Nhan, Ho Nguyen Anh Tuan

**Affiliations:** Pham Ngoc Thach University of Medicine, Ho Chi Minh City, Vietnam

**Keywords:** HIV stigma, HIV Stigma Scale, HSS-12, Psychometric properties, Reliability, Validity

## Abstract

**Background:**

HIV-related stigma remains a major psychosocial challenge affecting mental health, healthcare engagement, and quality of life among people living with HIV. Reliable and culturally appropriate brief instruments are essential for stigma assessment in both research and clinical practice; however, validated Vietnamese measures remain limited. This study aimed to evaluate the psychometric properties of the Vietnamese version of the 12-item HIV Stigma Scale (HSS-12).

**Methods:**

A cross-sectional study was conducted among 245 people living with HIV receiving antiretroviral therapy at a primary healthcare facility in Ho Chi Minh City, Vietnam. Internal consistency was assessed using Cronbach’s alpha. Test–retest reliability was evaluated in a subsample of 56 participants over a 4-week interval using the intraclass correlation coefficient (ICC). Construct validity was examined using confirmatory factor analysis (CFA), comparing a hypothesized four-factor model with a one-factor alternative model. Convergent validity was assessed through correlations with depressive symptoms (Patient Health Questionnaire-9; PHQ-9) and perceived social support (Multidimensional Scale of Perceived Social Support; MSPSS).

**Results:**

The Vietnamese HSS-12 demonstrated good internal consistency (Cronbach’s alpha = 0.85) and excellent test–retest reliability (ICC = 0.95). CFA supported a four-factor structure with improved model fit after allowing correlations between item residuals (CFI = 0.962; TLI = 0.945; RMSEA = 0.064; SRMR = 0.050), whereas the one-factor model showed poor fit. Convergent validity was supported by positive correlations between HSS-12 scores and PHQ-9 scores, and negative correlations with MSPSS scores, except for the disclosure concerns subscale.

**Conclusions:**

The Vietnamese version of the HSS-12 is a reliable and valid instrument for assessing perceived HIV-related stigma among people living with HIV in Vietnam. This brief measure may facilitate standardized stigma assessment in psychological research and routine care settings.

## Background

HIV remains a major global public health concern, with approximately 40.8 million people living with HIV worldwide in 2024 [1]. Although advances in antiretroviral therapy (ART) have transformed HIV into a manageable chronic condition, many people living with HIV continue to face substantial social barriers. Among these, HIV-related stigma is considered a central challenge, profoundly affecting physical health, mental health, and quality of life [2, 3].

The concept of stigma was originally described by Erving Goffman as an attribute that discredits an individual, leading to social devaluation, exclusion, and discrimination [4]. In the context of HIV, stigma is commonly conceptualized as a multidimensional phenomenon encompassing three closely related mechanisms: enacted stigma, anticipated stigma, and internalized stigma. Enacted stigma refers to experiences of discrimination or unfair treatment faced by people living with HIV from family members, communities, or healthcare settings. Anticipated stigma reflects individuals’ fears or expectations of being stigmatized in the future if their HIV status is disclosed. In contrast, internalized stigma captures the extent to which individuals accept and internalize society’s negative beliefs about HIV, which can adversely affect self-esteem, mental health, and engagement in health-related behaviors [5].

Numerous studies have demonstrated that HIV-related stigma is closely associated with delayed HIV testing, reduced adherence to ART, limited access to healthcare services, and decreased willingness to disclose HIV status to sexual partners or family members [3, 5–7]. Consequently, accurate and reliable measurement of HIV-related stigma among people living with HIV is crucial for research, clinical practice, and the design of effective stigma-reduction interventions.

Several instruments have been developed to assess HIV-related stigma [8]. Among these, the HIV Stigma Scale developed by Berger et al. [9] is one of the most widely used instruments, as it captures multiple dimensions of stigma, including personalized stigma, disclosure concerns, negative self-image, and concerns about public attitudes. However, the original 40-item HIV Stigma Scale has several limitations, including its length, which may impose a considerable respondent burden, and the presence of items that load onto multiple subscales, potentially limiting the distinctiveness of the underlying dimensions and complicating the interpretation of subscale scores [10, 11].

To address these limitations, Reinius et al. [12] developed a 12-item short version of the scale (HSS-12), retaining the four core dimensions of HIV-related stigma while reducing redundancy and improving feasibility for use in research and routine care. The HSS-12 has been validated in multiple countries and populations of people living with HIV, including Sweden [12], Brazil [13], Kenya [14], Portugal [11], Peru [15], and Iran [16]. Across these studies, the scale has generally demonstrated acceptable to good internal consistency [11–16], and studies that assessed temporal stability also reported good to excellent test–retest reliability [14, 16]. In addition, psychometric evaluations across different linguistic and sociocultural contexts have consistently supported its four-factor structure, with evidence of construct validity and expected associations with related psychosocial outcomes such as depression, anxiety, suicidal ideation, and perceived social support [11, 14].

In Vietnam, HIV-related stigma remains a significant public health concern, shaped by sociocultural factors. According to the Vietnam Authority of HIV/AIDS Control, in 2024 there were 13,351 newly reported HIV infections, with over 245,000 people currently living with HIV nationwide [17]. Early studies suggest that stigma has been rooted in the association of HIV with so-called “social evils,” such as drug use and sex work, leading to moral judgments and discrimination against people living with HIV [18, 19]. More recent evidence indicates that people living with HIV continue to experience stigma across multiple settings, including family, community, and healthcare environments. These experiences encompass both perceived stigma, manifested as internalized shame and concerns about negative social judgment, as well as enacted stigma, manifested as social avoidance in interpersonal relationships and differential treatment within healthcare settings, including ART clinics [20–22].

Studies examining the relationship between culture and HIV-related stigma suggest that both the level and expression of stigma vary across cultural contexts, and that factors such as social norms, moral values, and family structures play an important role in shaping stigma [5, 23]. In this context, in Vietnam, which is a collectivist society, stigma extends beyond the individual to affect family members through stigma by association, reflecting the importance of family reputation. As a result, individuals and families may seek to conceal HIV status to avoid social dishonor, which may delay access to healthcare services and social support [24, 25].

Against this background, accurate and consistent measurement of HIV-related stigma is essential for standardized assessment in research and clinical practice, facilitating routine stigma monitoring, and supporting the evaluation of stigma-reduction interventions. However, existing studies in Vietnam have used heterogeneous measurement tools, limiting comparability across studies and constraining the evaluation of intervention outcomes. To date, no validated Vietnamese version of the HSS-12 is available. This underscores the need to translate, culturally adapt, and evaluate the psychometric properties of the HSS-12 to ensure its conceptual relevance and measurement validity in the Vietnamese context. Therefore, this study aimed to translate and culturally adapt the HSS-12 into Vietnamese and to evaluate its reliability and validity among people living with HIV receiving ART at a primary healthcare facility in Ho Chi Minh City, Vietnam.

## Methods

### Study design and setting

A cross-sectional study was conducted from May to June 2025 at the Substance Use and HIV/AIDS Counseling and Treatment Department of District 10 Medical Center in Ho Chi Minh City, Vietnam.

District 10 Medical Center is a public primary healthcare facility providing preventive and basic curative services. It plays a key role in implementing national HIV/AIDS programs, including HIV testing, counseling, and antiretroviral therapy (ART) at the community level. As a district-level facility, it delivers long-term HIV care and follow-up, particularly for stable patients receiving ART. At the time of the study, the center was providing ART to approximately 2,000 people living with HIV, representing a substantial outpatient HIV cohort in Ho Chi Minh City, and thus constituted an appropriate setting for participant recruitment and psychometric evaluation of the Vietnamese version of the HSS-12.

### Participants

The study population consisted of people living with HIV who were receiving ART at District 10 Medical Center during the study period. Inclusion criteria were: (i) aged 18 years or older; (ii) having a confirmed diagnosis of HIV and currently receiving ART at the study site; (iii) ability to communicate in and read Vietnamese; and (iv) provision of written informed consent. Exclusion criteria included: (i) the presence of severe mental disorders or cognitive impairment that could affect the ability to complete the questionnaire; and (ii) missing or incomplete responses to one or more HSS-12 items.

### Sample size and sampling

The target sample size was determined a priori to ensure an adequate sample for confirmatory factor analysis (CFA). Commonly cited guidelines suggest that a sample size of approximately 200 is acceptable for factor analysis [26]. For test–retest reliability, the minimum sample size was calculated using Bonett’s method for estimating the intraclass correlation coefficient (ICC) with desired precision [27]. Assuming an expected ICC of 0.88 [16], a 95% confidence interval half-width of 0.06, and two administrations (k = 2), the required minimum sample size was 56 participants.

Participants were recruited using convenience sampling during routine clinic visits from 15 May to 15 June 2025. At the study site, patients receiving ART attended scheduled follow-up visits at intervals ranging from 2 to 12 weeks, depending on treatment status. For patients newly initiating ART, follow-up visits were typically scheduled after two weeks to monitor treatment response and potential adverse effects, whereas clinically stable patients were generally followed up on a monthly basis. In some cases, longer refill intervals (1.5 to 3 months) were provided based on individual circumstances. Given that approximately 2,000 people living with HIV were receiving ART at the center, a substantial proportion of patients were accessible during the study period.

Eligible patients attending routine clinic visits during the study period were invited to participate, with treating physicians assisting in facilitating initial contact. Participant selection was based on predefined eligibility criteria. A total of 250 patients were approached, of whom 245 agreed to participate and 5 declined, yielding a response rate of 98.0%. A total of 245 participants with complete data were included in the final analysis.

For the assessment of test–retest reliability, a subsample of 56 participants completed the HSS-12 a second time between 16 and 30 June 2025, corresponding to a 4-week interval after the initial assessment.

### Measures

Standardized self-report instruments were used in this study, including the 12-item HIV Stigma Scale (HSS-12), the Multidimensional Scale of Perceived Social Support (MSPSS), and the Patient Health Questionnaire–9 (PHQ-9).

#### HIV stigma scale–12 (HSS-12)

The 12-item HIV Stigma Scale (HSS-12), developed by Reinius et al. [12] based on the original HIV Stigma Scale by Berger et al. [9], was used to assess perceived HIV-related stigma. The scale comprises four dimensions: personalized stigma, disclosure concerns, negative self-image, and concerns about public attitudes. Each item is rated on a 4-point Likert scale ranging from 1 (“strongly disagree”) to 4 (“strongly agree”). The total HSS-12 score was calculated by summing responses across all 12 items (range: 12–48), with higher scores indicating higher levels of perceived HIV-related stigma. Subscale scores range from 3 to 12 [12].

#### Multidimensional scale of perceived social support (MSPSS)

The MSPSS is a 12-item self-report instrument designed to assess perceived social support from three sources: family, friends, and significant others, with four items per subscale [28]. Each item is rated on a 7-point Likert scale ranging from 1 (“strongly disagree”) to 7 (“strongly agree”). Subscale scores were calculated as the mean of the corresponding four items, and the total score was computed as the mean of all 12 items (range: 1–7), with higher scores indicating higher levels of perceived social support. The MSPSS has been translated into Vietnamese and validated in Vietnam, demonstrating good reliability and validity across different populations [29].

#### Patient Health Questionnaire–9 (PHQ-9)

The PHQ-9 is a 9-item self-report instrument used to assess depressive symptom severity over the preceding two weeks [30]. Items are rated on a 4-point Likert scale (0 = “not at all” to 3 = “nearly every day”), yielding a total score ranging from 0 to 27, with higher scores indicating greater depressive symptom severity. The PHQ-9 has been validated in Vietnam, demonstrating good internal consistency (Cronbach’s alpha = 0.88) [31].

### Procedure

**Fig. 1:**
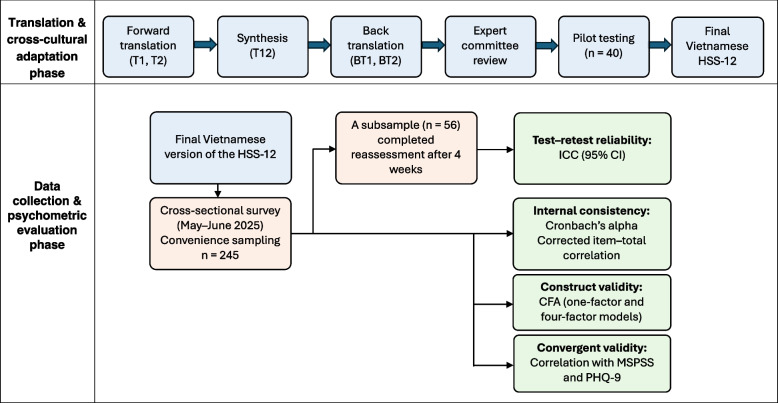
Overview of the translation, cross-cultural adaptation, and psychometric evaluation process of the Vietnamese HSS-12

An overview of the translation, cross-cultural adaptation, and psychometric evaluation workflow is presented in Fig. 1.

#### Stage 1: translation and cross-cultural adaptation

The short version of the HIV Stigma Scale (HSS-12) was translated into Vietnamese following the cross-cultural adaptation guidelines proposed by Beaton et al. [32], with an emphasis on conceptual equivalence. The process included independent forward translation by two bilingual translators who were native Vietnamese speakers, synthesis of the translated versions, back-translation into English by two independent translators blinded to the original scale, expert committee review, and development of a pre-final version. Permission to translate and use the scale was obtained from the original author prior to the translation process.

#### Stage 2: pilot study

The pre-final Vietnamese version of the HSS-12 was pilot-tested among 40 people living with HIV receiving ART at another healthcare facility in Ho Chi Minh City. Participants were asked to comment on the clarity, comprehensibility, and cultural relevance of the items, as well as any difficulties encountered during completion. Overall, participants reported that the items were clear and culturally appropriate, and no major issues were identified during pilot testing. Therefore, no further modifications were made, and the pre-final version was retained as the final Vietnamese HSS-12.

#### Stage 3: reliability and validity evaluation

The finalized Vietnamese version of the HSS-12 was administered in the main cross-sectional study conducted at District 10 Medical Center. Data were collected during routine follow-up visits after informed consent was obtained. To assess test–retest reliability, a subsample of participants (n = 56) completed the HSS-12 again after a 4-week interval. The collected data were used to evaluate internal consistency, test–retest reliability, construct validity, and convergent validity of the scale.

### Data collection

Data collection was conducted at the Substance Use and HIV/AIDS Counseling and Treatment Department at District 10 Medical Center during the study period. Eligible participants attending routine follow-up visits during the study period were invited to participate. After receiving information about the study objectives, procedures, and ethical considerations, those who agreed to participate provided written informed consent.

Data were collected using self-administered questionnaires, which included sociodemographic characteristics, clinical information, and three standardized instruments: the HSS-12, MSPSS, and PHQ-9. Clinical and treatment-related data, including time since HIV diagnosis, duration of ART, and most recent CD4 cell counts, were extracted from medical records using a structured extraction form.

To assess test–retest reliability of the HSS-12, a subsample of participants (n = 56) completed the HSS-12 again 4 weeks after the initial assessment. All questionnaires and extracted data were anonymized using unique study identification codes to ensure confidentiality while allowing linkage between the two data collection time points.

### Data analysis

Data were entered, cleaned, and analyzed using Stata version 14.0. No missing data were observed for HSS-12 items among the participants included in the final analysis. Descriptive statistics were used to summarize participants’ characteristics, with frequencies and percentages for categorical variables, and means with standard deviations or medians with interquartile ranges (IQR) for continuous variables, as appropriate.

The internal consistency of the HSS-12 and its subscales was assessed using Cronbach’s alpha and corrected item–total correlation coefficients. A Cronbach’s alpha value of ≥ 0.70 was considered acceptable, while values ≥ 0.80 indicated good internal consistency [33]. Corrected item–total correlations of ≥ 0.30 were considered satisfactory [33]. Test–retest reliability of the HSS-12 was evaluated using a two-way mixed-effects, consistency, single-measure intraclass correlation coefficient [ICC(3,1)] with 95% confidence intervals, based on data collected after a 4-week interval. ICC values > 0.75 were interpreted as good reliability, and values ≥ 0.90 indicated excellent reliability [34].

The suitability of the data for factor analysis was assessed using the Kaiser–Meyer–Olkin (KMO) measure and Bartlett’s test of sphericity [35]. Construct validity was examined using confirmatory factor analysis (CFA). Given the established four-factor structure of the HSS-12, CFA was conducted to evaluate the fit of the original model in the Vietnamese context. Both the hypothesized four-factor model and a one-factor alternative model were tested. CFA was performed using maximum likelihood estimation. Model modifications, if necessary, were informed by theoretical considerations and guided by modification indices, with adjustments limited to correlated residuals between items within the same factor, particularly for items with similar wording or conceptual overlap, and without altering the underlying factor structure. Although the chi-square (χ2) test was used to assess overall model fit, given its sensitivity to sample size, additional fit indices were used to provide a comprehensive evaluation of model fit. Specifically, Comparative Fit Index (CFI) and Tucker–Lewis Index (TLI) values > 0.90 were considered indicative of acceptable fit, while values ≥ 0.95 indicated excellent fit [36]. Root Mean Square Error of Approximation (RMSEA) and Standardized Root Mean Square Residual (SRMR) values < 0.08 were considered acceptable [37].

Convergent validity was assessed using Pearson’s correlation coefficients between the HSS-12 total and subscale scores and the MSPSS and PHQ-9 scores. Negative correlations with MSPSS and positive correlations with PHQ-9 were hypothesized. A two-sided p value < 0.05 was considered statistically significant.

## Results

### Baseline characteristics of participants

Table 1 presents the demographic and clinical characteristics of the 245 participants included in the study. The mean age was 33.8 ± 8.2 years, and most participants were male (83.7%). Approximately three-quarters had completed at least a high school education (75.0%). Most participants were single (74.3%). The median time since HIV diagnosis was 4 years (IQR: 3–9), and the median duration of ART was also 4 years (IQR: 3–9). Nearly half of the participants (47.1%) had a CD4 cell count of ≥ 500 cells/mm3. A total of 56 participants completed the retest assessment, and the characteristics of this subgroup were generally similar to those of the full sample.

**Table 1:** Baseline Characteristics of Participants

Characteristics	All participants (n = 245)	Retest participants (n = 56)
Age (years)	33.8 ± 8.2	32.7 ± 8.2
Gender		
Male	205 (83.7)	48 (85.7)
Female	37 (15.1)	7 (12.5)
Other	3 (1.2)	1 (1.8)
Education level		
Under primary school	10 (4.1)	1 (1.8)
Primary school	8 (3.3)	0 (0.0)
Secondary school	43 (17.6)	7 (12.5)
High school	59 (24.0)	18 (32.1)
Higher education	125 (51.0)	30 (53.6)
Marital status		
Single	182 (74.3)	42 (75.0)
Married	43 (17.6)	10 (17.8)
Separated/Divorced/Widowed	15 (6.1)	2 (3.6)
Living with partner	5 (2.0)	2 (3.6)
Duration of HIV infection (years)	4 (3–9)	4 (3–9)
Duration of ART (years)	4 (3–9)	4 (3–9)
CD4 count (cells/mm^3^)		
≤ 200	22 (9.0)	2 (3.6)
201–349	40 (16.4)	7 (12.5)
350–499	67 (27.5)	14 (25.0)
≥ 500	115 (47.1)	33 (58.9)

Values are presented as mean ± standard deviation or median (interquartile range) for continuous variables, and number (percentage) for categorical variables. CD4 data were available for 244 of 245 participants because of missing laboratory results

*Abbreviations: HIV *Human immunodeficiency virus,* ART *Antiretroviral therapy,* CD4 *Cluster of differentiation 4 T-lymphocyte count

### Reliability of the Vietnamese version of the HSS-12

As shown in Table 2, the Vietnamese version of the HSS-12 demonstrated good internal consistency, with Cronbach’s alpha coefficients ranging from 0.690 to 0.838 across the four subscales and 0.849 for the total scale. Corrected item–total correlation coefficients ranged from 0.309 to 0.677, indicating acceptable to good item discrimination, with all items exceeding the commonly accepted threshold of 0.30. Cronbach’s alpha if item deleted ranged from 0.826 to 0.853. No item deletion produced a substantial improvement in the total Cronbach’s alpha. Although deletion of Item 8 would slightly increase the total alpha from 0.849 to 0.853, this difference was minimal, and the corrected item–total correlation for Item 8 remained above the acceptable threshold of 0.30. In addition, Item 8 directly reflects selective disclosure of HIV status, a core aspect of the disclosure concerns subscale. Therefore, all 12 items were retained. Test–retest reliability assessed in 56 participants over a 4-week interval was excellent, with ICC values of 0.90–0.93 across subscales and 0.95 (95% CI: 0.91–0.97) for the total HSS-12 score.

**Table 2:** Reliability of the HSS-12 and its subscales

Item number and description	Mean ± SD	Item–total correlation	Cronbach’s alpha if item deleted	Cronbach’s alpha	ICC (95% CI)
Personalized stigma	6.7 ± 2.0			0.838	0.93 (0.89–0.96)
Item 10. Some people avoid touching me once they know I have HIV	2.4 ± 0.8	0.677	0.826		
Item 11. People I care about stopped calling after learning I have HIV	2.2 ± 0.8	0.523	0.838		
Item 12. I have lost friends by telling them I have HIV	2.1 ± 0.7	0.581	0.834		
Disclosure concerns	8.5 ± 2.0			0.729	0.90 (0.84–0.94)
Item 3. Telling someone I have HIV is risky	2.7 ± 0.9	0.534	0.837		
Item 4. I work hard to keep my HIV a secret	2.9 ± 0.8	0.441	0.843		
Item 8. I am very careful who I tell that I have HIV	2.9 ± 0.8	0.309	0.853		
Concerns about public attitudes	6.7 ± 2.1			0.817	0.90 (0.83–0.94)
Item 6. People with HIV are treated like outcasts	2.2 ± 0.8	0.622	0.830		
Item 7. Most people believe that a person who has HIV is dirty	2.1 ± 0.8	0.603	0.832		
Item 9. Most people are uncomfortable around someone with HIV	2.4 ± 0.8	0.601	0.831		
Negative self-image	6.7 ± 1.9			0.690	0.92 (0.87–0.95)
Item 1. I feel guilty because I have HIV	2.3 ± 0.9	0.406	0.847		
Item 2. People’s attitudes about HIV make me feel worse about myself	2.1 ± 0.8	0.448	0.843		
Item 5. I feel I am not as good a person as others because I have HIV	2.2 ± 0.8	0.508	0.839		
HSS-12 total	28.6 ± 6.0			0.849	0.95 (0.91–0.97)

Values are presented as mean ± SD for the HSS-12 subscale and total scale scores. Corrected item–total correlations and Cronbach’s alpha if item deleted were calculated for the total HSS-12 scale. ICC was calculated for the total HSS-12 score and subscale scores among retest participants (n = 56) over a 4-week interval

*Abbreviations: HSS-12 *HIV Stigma Scale–12,* SD *Standard deviation,* ICC *Intraclass correlation coefficient,* CI *Confidence interval

### Construct validity (CFA) of the Vietnamese version of the HSS-12

The KMO value was 0.825, and Bartlett’s test of sphericity was statistically significant (χ2 = 1219.301, df = 66, p < 0.001), indicating suitability for factor analysis.

Confirmatory factor analysis (CFA) was conducted to examine the hypothesized four-factor structure of the Vietnamese version of the HSS-12, including personalized stigma, disclosure concerns, concerns about public attitudes, and negative self-image. As shown in Table 3, the initial four-factor model demonstrated suboptimal fit to the data (χ2 = 164.95, df = 48, p < 0.001; χ2/df = 3.44; RMSEA = 0.100, 90% CI: 0.083–0.117; CFI = 0.901; TLI = 0.864; SRMR = 0.066). After allowing three theoretically justified within-factor residual correlations, the four-factor model showed substantially improved and acceptable fit (χ2 = 89.60, df = 45, p < 0.001; χ2/df = 1.99; RMSEA = 0.064, 90% CI: 0.044–0.083; CFI = 0.962; TLI = 0.945; SRMR = 0.050). This improvement was also reflected in the χ2/df ratio, which decreased from 3.44 in the initial four-factor model to 1.99 in the final model. Specifically, residual correlations were specified between Items 11 and 12 within the personalized stigma subscale, Items 4 and 8 within the disclosure concerns subscale, and Items 6 and 7 within the concerns about public attitudes subscale. The corresponding standardized residual correlations were 0.52, 0.29, and 0.45, respectively. The final four-factor model also yielded the lowest AIC value among the compared models, indicating a better balance between model fit and parsimony. In contrast, the one-factor model demonstrated poor fit (χ2 = 436.19, df = 54, p < 0.001; χ2/df = 8.08; RMSEA = 0.170, 90% CI: 0.155–0.185; CFI = 0.677; TLI = 0.605; SRMR = 0.107), supporting the multidimensional structure of the HSS-12.

**Table 3:** Model fit indices for confirmatory factor analysis of the Vietnamese version of the HSS-12

	**One-factor model**	**Four-factor model (initial)**	**Four-factor model with correlated residuals**
χ^2^ (df)	436.19 (54), p < 0.001	164.95 (48), p < 0.001	89.60 (45), p < 0.001
χ^2^/df	8.08	3.44	1.99
RMSEA (90% CI)	0.170 (0.155–0.185)	0.100 (0.083–0.117)	0.064 (0.044–0.083)
SRMR	0.107	0.066	0.050
CFI	0.677	0.901	0.962
TLI	0.605	0.864	0.945
AIC	6335.72	6076.48	6007.12

The final four-factor model included three within-factor correlated residuals between Items 11–12, Items 4–8, and Items 6–7. No cross-factor residual correlations were specified

*Abbreviations: RMSEA *Root Mean Square Error of Approximation,* SRMR *Standardized Root Mean Square Residual*, CFI *Comparative Fit Index*, TLI *Tucker–Lewis Index*, AIC *Akaike Information Criterion

**Fig. 2:**
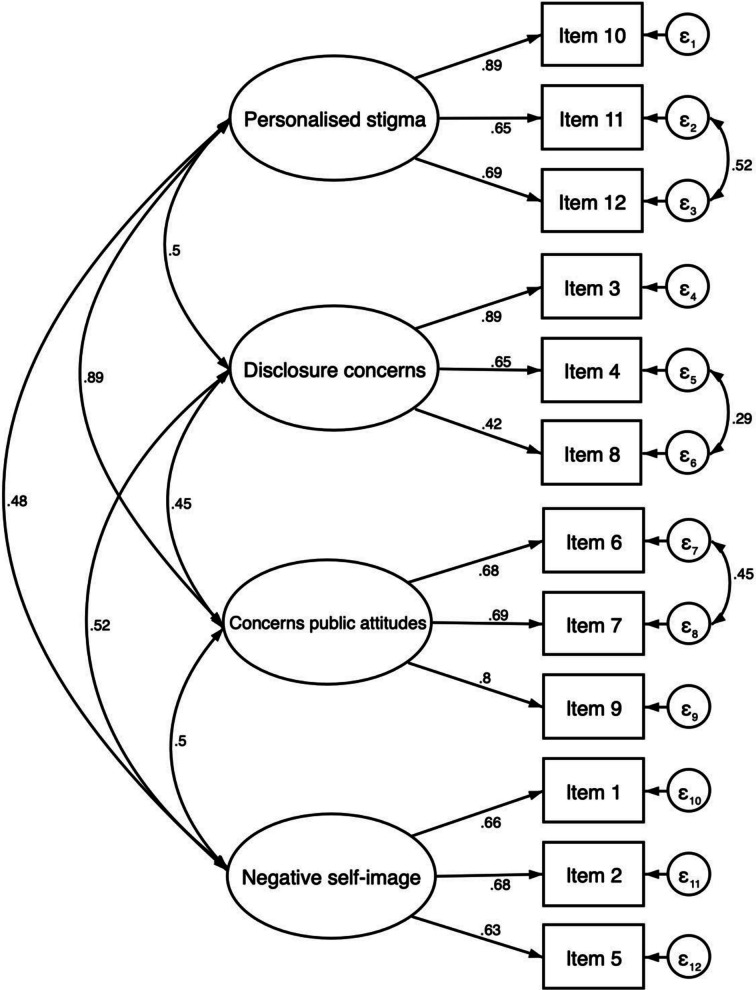
Standardized CFA model for the four-factor model with correlated residuals of the Vietnamese HSS-12. Note: Single-headed arrows indicate standardized factor loadings; curved double-headed arrows between latent factors indicate inter-factor correlations; curved double-headed arrows between residuals indicate within-factor residual correlations

The standardized CFA model is presented in Fig. 2. All items demonstrated adequate standardized factor loadings (all ≥ 0.40) on their respective latent factors, and the four stigma domains showed moderate to strong inter-factor correlations, ranging from 0.45 to 0.89.

### Convergent validity of the Vietnamese version of the HSS-12

As shown in Table 4, convergent validity of the Vietnamese version of the HSS-12 was supported by correlations with external measures in the expected directions. All four HSS-12 subscales and the total score were positively correlated with depressive symptoms measured by the PHQ-9 (r = 0.163–0.279, *p* < 0.05). In contrast, personalized stigma, concerns about public attitudes, negative self-image, and the total HSS-12 score were negatively correlated with perceived social support measured by the MSPSS (r = − 0.186 to − 0.265, *p* < 0.01). Disclosure concerns showed a weak and non-significant correlation with MSPSS (r = − 0.097, p > 0.05).

**Table 4:** Convergent validity of the Vietnamese version of the HSS-12

	**Personalized stigma**	**Disclosure concerns**	**Concerns about public attitudes**	**Negative self-image**	**HSS-12 total**
PHQ-9	0.221***	0.163*	0.192**	0.253***	0.279***
MSPSS	** − **0.254***	** − **0.097	** − **0.247***	** − **0.186**	** − **0.265***

Values are Pearson’s correlation coefficients (r)

*Abbreviations: PHQ-9 *Patient Health Questionnaire-9.* HSS-12 *HIV Stigma Scale–12,* MSPSS *Multidimensional Scale of Perceived Social Support

^*^p < 0.05; **p < 0.01; ***p < 0.001

## Discussion

This study provides initial evidence supporting the reliability and validity of the Vietnamese HSS-12 among people living with HIV receiving ART in Vietnam. Given the lack of standardized Vietnamese instruments for HIV-related stigma assessment, the HSS-12 may serve as a brief and feasible tool for research and routine care. Overall, the scale demonstrated satisfactory psychometric performance in this setting.

The Vietnamese version of the HSS-12 demonstrated good internal consistency, with a Cronbach’s alpha of 0.85 for the total scale and values ranging from 0.69 to 0.84 across the subscales. These values fall within acceptable thresholds and are consistent with findings from the original study by Reinius et al. [12], as well as validation studies conducted in Brazil [13], Kenya [14], Peru [15], and Iran [16]. Although some previous studies have reported Cronbach’s alpha values below 0.70 for certain subscales, particularly in studies with small sample sizes [11, 16], the results of the present study indicate that the Vietnamese version of the HSS-12 exhibits satisfactory internal consistency within the current study context. In addition, test–retest reliability over a 4-week interval was excellent for the total HSS-12 score (ICC = 0.95), indicating excellent temporal stability. This finding is comparable to, and within the range of, those reported in studies conducted in Kenya (ICC = 0.92) [14] and Iran (ICC = 0.88) [16].

Confirmatory factor analysis (CFA) supported the hypothesized four-factor structure of the HSS-12, comprising personalized stigma, disclosure concerns, concerns about public attitudes, and negative self-image. Although the chi-square test was statistically significant, other model fit indices, including the CFI, TLI, RMSEA, and SRMR, indicated acceptable to good model fit, consistent with psychometric research recognizing the sensitivity of the chi-square statistic to sample size [36, 38]. After allowing three theoretically and conceptually justified within-factor residual correlations, the four-factor model demonstrated substantially improved fit. Specifically, Items 11 ("People I care about stopped calling after learning I have HIV") and 12 ("I have lost friends by telling them I have HIV") both describe interpersonal rejection or relationship loss following HIV disclosure; Items 4 ("I work hard to keep my HIV a secret") and 8 ("I am very careful who I tell that I have HIV") both reflect concealment and selective disclosure of HIV status; and Items 6 ("People with HIV are treated like outcasts") and 7 ("Most people believe that a person who has HIV is dirty") both capture negative public attitudes toward people living with HIV by referring to how people with HIV are perceived or treated by the public. Because all residual correlations were specified within the original subscales, these modifications did not alter the four-factor structure of the HSS-12. However, they suggest that some item pairs may share specific meaning beyond their common latent construct, which should be considered when interpreting subscale scores. Future studies should examine whether the same residual correlation pattern is replicated in other Vietnamese samples and should test measurement invariance across gender, geographic regions, and cultural contexts before making strong between-group or cross-cultural comparisons. Moreover, the poor fit of the one-factor model further supported the multidimensional structure of the HSS-12. In the final four-factor model, all items exhibited acceptable standardized factor loadings, indicating that each item adequately represented its corresponding latent construct. In addition, moderate to strong inter-factor correlations (r = 0.45–0.89) suggest that these domains are related yet distinct, reinforcing the multidimensional nature of perceived HIV-related stigma. These findings are consistent with previous validation studies conducted in Sweden [12], Brazil [13], Portugal [11], and Peru [15], and support the structural validity of the four-factor model in the Vietnamese context.

Convergent validity of the Vietnamese version of the HSS-12 was supported by the expected pattern of associations with external psychological measures. Higher perceived HIV-related stigma was positively associated with depressive symptoms, as measured by the PHQ-9, and negatively associated with perceived social support, as measured by the MSPSS. Although the observed correlations were small to moderate in magnitude, their directions were consistent with theoretical expectations and findings from previous international studies. Prior meta-analyses have consistently demonstrated positive associations between HIV-related stigma and depression or psychological distress, as well as negative associations with social support among people living with HIV [39, 40]. Similar patterns have also been reported in validation studies of the HSS-12 conducted in different cultural contexts, including Portugal [11]. However, one exception was the weak and non-significant association between disclosure concerns and perceived social support, which is noteworthy. This finding may partly reflect the sensitive and strategic nature of HIV disclosure. In the Vietnamese context, where HIV-related stigma is shaped by family and community relationships, people living with HIV may remain concerned about disclosure even when they perceive support from family members, friends, or significant others [24, 25]. Selective nondisclosure may function as a coping strategy to avoid anticipated stigma, protect existing relationships, and prevent potential negative consequences for the family [25, 41]. Overall, these findings provide preliminary evidence for the convergent validity of the Vietnamese HSS-12.

The present findings should be interpreted in light of several limitations. First, the study sample was drawn from a single urban ART clinic using convenience sampling; therefore, caution is warranted when generalizing the findings to the broader population of people living with HIV in Vietnam. The predominance of male participants (83.7%) is broadly consistent with recent national surveillance data reported by the Vietnam Ministry of Health, which indicated that 84.3% of newly detected HIV cases in 2023 were male and that sexual transmission accounted for 80.8% of newly detected cases [42]. However, newly detected cases may not reflect the demographic profile of all people living with HIV or all ART patients nationwide. Thus, although the gender distribution of the sample is broadly aligned with recent national trends in newly detected cases, the underrepresentation of women, transgender people, other key populations, and people living in rural areas should be considered when interpreting the findings. Gender imbalance and the urban setting may also influence stigma perception and psychometric performance, as stigma experiences may differ according to gender norms, family roles, community visibility, service access, and social support [43, 44]. These contextual differences may affect item endorsement, factor loadings, and the generalizability of reliability and validity estimates. Future studies should validate the Vietnamese HSS-12 in more diverse samples and test measurement invariance across gender, key populations, and geographic regions.

Second, physician-assisted recruitment may have introduced some degree of selection bias, although participant recruitment was based on predefined eligibility criteria and was applied consistently throughout the study period. Furthermore, because all stigma-related measures were self-reported, responses may also be influenced by social desirability bias.

Another limitation is that convergent validity was assessed using related psychosocial measures (MSPSS and PHQ-9), rather than stigma-specific external criteria. Therefore, the observed correlations should be interpreted as preliminary and indirect evidence of convergent validity. Future validation studies should include stigma-specific constructs, such as enacted stigma, anticipated stigma, internalized stigma, HIV-related discrimination, or healthcare-related stigma, to strengthen validity evidence for the Vietnamese HSS-12.

## Conclusions

In conclusion, the findings of this study indicate that the Vietnamese version of the HSS-12 demonstrates good reliability and validity for assessing perceived HIV-related stigma among people living with HIV in Vietnam. The scale exhibited good internal consistency, excellent test–retest reliability, and acceptable evidence of construct and convergent validity. The Vietnamese HSS-12 may be used in research and clinical settings to facilitate standardized stigma assessment and routine stigma monitoring, and to support the development and evaluation of stigma-reduction interventions.

## Data Availability

The datasets used and/or analysed during the current study are not publicly available due to concerns regarding participant confidentiality but are available from the corresponding author on reasonable request.

## Abbreviation

AIDS: Acquired Immunodeficiency Syndrome
ART: Antiretroviral therapy
CFA: Confirmatory factor analysis
CFI: Comparative Fit Index
CI: Confidence interval
HIV: Human immunodeficiency virus
HSS-12: 12-Item HIV Stigma Scale
ICC: Intraclass correlation coefficient
MSPSS: Multidimensional Scale of Perceived Social Support
PHQ-9: Patient Health Questionnaire-9
RMSEA: Root Mean Square Error of Approximation
SD: Standard deviation
SRMR: Standardized Root Mean Square Residual
TLI: Tucker–Lewis Index
